# The Effect of Extended Dissection of Lymph Nodes (D2plus) with Gastrectomy on the Clinical and Oncological Outcomes in Gastric Cancer Patients, Compared to a Standard Dissection (D2)

**DOI:** 10.3390/medicina61071284

**Published:** 2025-07-16

**Authors:** Sahar Lazari, Muhammad Masalha, Forat Swaid, Walid Shalata, Gideon Sroka, Weam Waked, Abed Agbarya

**Affiliations:** 1 Department of Pediatric Surgery, Rambam Health Care Campus, 8 HaAliya HaShniya Street, Haifa 3109601, Israel; saharlazari86@gmail.com; 2 Department of Surgery, Tzafon Medical Center, Poriya 1528001, Israel; m7md.massal7a@gmail.com (M.M.); fswaid@tzmc.gov.il (F.S.); 3 The Legacy Heritage Cancer Center, Dr. Larry Norton Institute, Soroka Medical Center, Ben-Gurion University, Beer Sheva 84105, Israel; walid.shalata@gmail.com; 4 Faculty of Health Sciences, Ben Gurion University of the Negev, Beer Sheva 84105, Israel; 5 Department of Surgery, Bnai-Zion Medical Center, 47 Eliyahu Golomb Avenue, Haifa 3339419, Israel; gideon.sroka@b-zion.org.il (G.S.); weamwaked@gmail.com (W.W.); 6 Institute of Oncology, Bnai-Zion Medical Center, 47 Eliyahu Golomb Avenue, Haifa 3339419, Israel; 7 The Ruth and Bruce Rappaport Faculty of Medicine, Technion, Israel Institute of Technology, 1 Efron Street, Haifa 3339101, Israel

**Keywords:** gastric cancer, surgery, dissection, lymph nodes, survival, recurrence, D2 lymphadenectomy, D2plus, extended, gastrectomy

## Abstract

*Background and Objectives*: Gastric cancer treatment of partial or complete gastrectomy includes lymph nodes dissection (D2) to remove microscopic lymph node metastases adjacent to the tumor. A more extensive approach, an extended dissection (D2plus) has recently been employed, which includes resection of the lymph nodes in the pancreatic and periportal areas. However, despite its potential benefits of longer survival for patients diagnosed with advanced cancer, there are increased risks due to surgical complications. The current study aims to examine the balance between clinical benefit and higher risks of the extended dissection approach versus standard dissection. *Materials and Methods*: This retrospective analysis of gastric cancer patients treated in Bnai-Zion medical center examined the survival rates, oncological outcomes, and complication rates according to medical records data files. *Results*: The D2plus group experienced increased postoperative complications rate (56% vs. 20.6% D2 group *p* = 0.005) with mean survival time, shorter than the D2 standard approach (2.07 years vs. 3.44 years *p* = 0.01). A higher number of lymph nodes was removed on average in the D2plus group (29.4 ± 11.2), but without statistical significance in comparison to the D2 group (22.6 ± 8.9, *p* = 0.013). D2plus patients had reduced disease recurrence rates (20% vs. 32.4% in D2 group *p* = 0.29). Weight loss of D2plus patients was noted for higher rates than the D2 group (40% vs. 17.6% *p* = 0.056. *Conclusions*: Our study provides preliminary insights into the comparison between D2 and D2plus dissection in a single-center Western cohort. However, significant baseline differences between groups, particularly age, gender, and histopathological characteristics, limit definitive conclusions. The findings should be interpreted as hypothesis-generating rather than practice-changing. Larger, prospective, multicenter studies with propensity score matching or randomized design are needed to definitively establish the optimal surgical approach for different patient subgroups.

## 1. Introduction

Gastric cancer is the fifth-most-common cancer among all types of cancer, but the fourth-leading cause of death from cancer [1]. The 5-year survival rates in cases of advanced disease range from 20% to 30% [2,3]. The risk of death from gastric cancer is mainly due to the late diagnosis and the limited ability to manage it in the advanced stages of the disease [4]. The main reason for the complexity of the treatment is the inability to diagnose the disease in the early stages due to vague symptoms, such as mild abdominal pain, a feeling of swelling, or general fatigue [3,5]. As a result, most patients are diagnosed when the tumor is already advanced and has spread to nearby lymph nodes or even distant organs; thus, the poor prognosis [6].

In an advanced stage of the disease, the most-common treatment is gastrectomy, an operation in which the stomach is partially or completely removed, combined with dissection of the lymph nodes in stations 1, 2, 3, 4, 5, 6, 7, 8a, 9, 10, 11p, and 12a adjacent to the tumor [7,8]. This dissection, called D2, is considered standard practice in most treatment centers in the West. The aim of the D2 technique, by removal of the lymph nodes in the peripheral area of the stomach, is to prevent the spread of microscopic metastases to nearby organs and reduce the risk of recurrence of the disease. However, this dissection is limited in scope and does not include more distant areas where there may be microscopic metastases. Therefore, in recent years, experts recommend the extension of the dissection to more distant lymph nodes [8].

A more extensive approach, extended dissection (D2plus), includes the removal of lymph nodes also in more distant areas such as the periportal area, pancreas, and liver (D2 stations and 8p, 12b, 13, and 14v [7,9,10]. The goal is to improve the chance of controlling the tumor and additional microscopic metastases that are not visible on the initial imaging tests [11]. The extended dissection (D2plus) technique is applied in many medical centers in East Asia, such as Japan and South Korea, and is considered the standard treatment for gastric cancer patients [9,12,13,14].

Studies conducted in East Asia indicated that extended dissection can lead to a significant improvement in survival rates, especially among patients in advanced stages of gastric cancer, in which the tumor often spreads to many distant lymph nodes [3,15]. In Eastern countries, the 5-year survival rate is higher in gastric cancer patients undergoing the D2 plus approach than standard dissection D2 [3]. These data substantiate the claim that extended dissection can offer an oncological advantage [11]. Moreover, a decrease in tumor recurrence rates was observed among patients with advanced disease that involve multiple lymph nodes.

Despite the potential benefits of extended dissection, there are also significant risks for patients undergoing this operating technique. Removal of additional lymph nodes in the areas of the pancreas and liver increases the risk of surgical complications, including intraoperative bleeding, postoperative infections, and pancreatic leaks, which are more common complications in patients undergoing extended dissection [15]. Recent studies have reported that performing an extended dissection in older Western patients or those with underlying diseases may increase the mortality rate due to these complications [16,17]. Therefore, some medical centers adopted the more conservative approach and favor the standard D2 dissection, for fear of increasing the complication rate [18,19], while in East Asia, the D2plus method is considered to have a significant advantage [13,20].

Unreached consensus for gastric cancer patients’ treatment due to different approaches that are applied around the world, and the questions raised about the use of D2plus procedure, call for further research to examine the balance between the oncological benefits of extended dissection and the surgical risks.

The current study aims to examine the advantages and disadvantages of extended dissection (D2plus) compared to standard dissection (D2) for gastric cancer patients, focusing on oncological outcomes, survival rates, the rate of surgical complications, and quality of life after surgery.

## 2. Materials and Methods

### 2.1. Study Design

The current retrospective study focuses on examining the outcome of extended dissection (D2plus) versus standard dissection (D2) among gastric cancer patients who underwent gastrectomy surgery at the Bnai Zion Medical Center between 2017 and 2024. The data collected from medical records files included clinical, oncological, pathological, and surgical characteristics of the patients.

### 2.2. The Study Population

**Figure 1:**
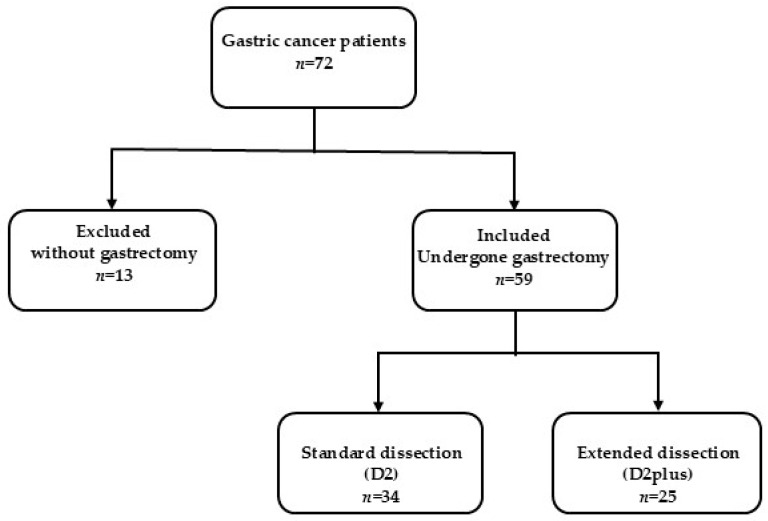
The study participant selection process at Bnai Zion Medical Center. Gastric cancer patients who underwent gastrectomy with standard or extended lymph node dissection were included. Patients without gastrectomy were excluded due to a very advanced disease with omental spread.

Initially, data were collected for 72 patients diagnosed with gastric cancer. However, 59 patients who underwent partial or complete gastrectomy between January 2017 and December 2024 at the Bnai Zion Medical Center were ultimately included. Thirteen patients were classified as being in very advanced stages with diffuse omental spread and, therefore, did not undergo gastrectomy surgery (Figure 1). The research population was divided into two main groups:

1. Standard dissection group (D2), which included 34 patients who underwent a standard dissection with the removal of the nearby lymph nodes in stations 1, 2, 3, 4, 5, 6, 7, 8a, 9, 10, 11p, and 12.
2. Extended dissection group (D2plus), which included 25 patients who underwent an extended dissection with the removal of regional and distant (pancreas, liver) lymph nodes in stations 1, 2, 3, 4, 5, 6, 7, 8a, 8p, 9, 10, 11p, 12a, 12b, 13, and 14v.

#### 2.2.1. Inclusion Criteria

Patients were at least 18 years old, diagnosed with gastric cancer according to clinical and pathological criteria, underwent gastrectomy by dissection (D2 or D2plus) between January 2017 and December 2024, and had availability of complete clinical information regarding the outcomes of the surgery, e.g., postoperative follow-up of at least 6 months after surgery.

#### 2.2.2. Exclusion Criteria

Patients who have undergone previous operations in the gastrointestinal system that affected the condition of the stomach, patients who suffered from other serious chronic diseases unrelated to gastric cancer (such as heart disease or liver failure), and/or patients lacking complete medical records data.

### 2.3. Surgery and Surgical Selection Criteria

All operations and procedures were conducted at the Bnai Zion Medical Center Department of Surgery, which specializes in Laparoscopy done by expert surgeons. Multi-disciplinary tumor board discussions for each case suggested an individual protocol concerning the surgery type. Bnai Zion Medical Center is a teaching hospital providing supervised programs for trainees.

The surgical approach (D2 vs. D2plus) was determined by multidisciplinary tumor board discussions considering multiple factors: tumor location, clinical staging (cT and cN), patient age and comorbidities, surgeon expertise, and institutional resources. D2plus dissection was generally favored for patients with suspected higher nodal involvement (cN+), tumors located in the upper stomach requiring extensive resection, and younger patients with good performance status who could tolerate the extended procedure. The decision was individualized for each patient, considering the balance between potential oncological benefit and surgical risk.

### 2.4. Data Collection

The data collected from the patients’ medical files included the following demographic variables: age, gender, and underlying diseases. The clinical and pathological parameters were recorded, e.g., the stage of the tumor (TNM) and the number of involved lymph nodes. In addition, oncological outcomes were gathered concerning the survival rates, surgical complications (intraoperative bleeding, inflammation, infections, pancreatic leaks), hospitalization after surgery, and the recurrence rate of the disease. Quality-of-life parameters were assessed during routine follow-up visits through clinical evaluation focusing on functional status, nutritional parameters (weight loss), and symptom assessment (pain). Pain was assessed using verbal pain scales (0–10) during clinic visits and documented in medical records. Weight loss was defined as >10% reduction from baseline weight documented at follow-up visits.

### 2.5. Outcome Definitions

Disease recurrence was defined as radiological, endoscopic, or histopathological evidence of gastric cancer recurrence (local, regional, or distant) identified during follow-up imaging studies (CT, MRI, or PET-CT) or clinical evaluation. All suspected recurrences were confirmed by multidisciplinary team review.

Overall survival was measured from the date of surgery to death from any cause or last follow-up. Disease-free survival was measured from the date of surgery to first evidence of recurrence or death, whichever occurred first.

### 2.6. Statistical Analysis

Descriptive statistics were calculated in terms of mean, standard deviation, median, and interquartile ranges. The normal distribution of continuous variables was analyzed by Kolmogorov-Smirnov test. As a result of this test, a *t*-test, or Mann-Whitney U test, was used to compare and examine differences between the two groups (D2 or D2plus). A Pearson chi-square test, or a Fisher exact test, was performed to assess independence between categorical variables. Kaplan-Meier curve was used to analyze time-to-event data (death). *p* < 0.05 was considered as significant. All the statistical analysis was done by SPSS software, version 28 (IBM, Armonk, NY, USA).

### 2.7. Ethics

The research was conducted in compliance, and in accordance, with the guidelines of the Bnai Zion Medical Center Ethics Committee, which approved the study design in accordance with the Declaration of Helsinki (Protocol BNZ-2024-0096). Patient consent was waived due to the retrospective nature of the study. All medical information used for the research remains confidential, and the research does not include personal identification of the patients.

## 3. Results

The current study evaluated the aspects related to extended dissection of the lymph nodes (D2plus) in comparison to standard dissection (D2), offered for gastric cancer patients by comparing survival rates, the rate of surgical complications, and the post-operative quality of life.

### 3.1. Patients’ Demographic Characteristics

The demographic analysis revealed significant baseline differences between groups that may influence survival outcomes. The mean age in the D2 group was significantly higher than that in the D2plus group (71.6 ± 9.7 years vs. 63.1 ± 16.2 years; *p* = 0.014). In addition, a significant gender difference was found between the groups. In the D2 group, the majority were men (70.6%), and in the D2plus group, there was a higher proportion of women (60%; *p* = 0.019). These demographic differences represent potential confounding factors that must be considered when interpreting survival comparisons between groups (Table 1).

#### 3.1.1. Histopathological Characteristics

Analysis of histopathological data revealed important differences between the two groups. The D2plus group had a significantly higher proportion of signet ring cell carcinoma (SRC) compared to the D2 group (40.0% vs. 20.6%). Specifically:

- D2 group: 23/34 patients (67.6%) had adenocarcinoma, 7/34 patients (20.6%) had SRC
- D2plus group: 9/25 patients (36.0%) had adenocarcinoma, 10/25 patients (40.0%) had SRC

This histological imbalance represents an important confounding factor that may influence survival outcomes, as SRC histology is associated with more aggressive behavior and poorer prognosis regardless of surgical approach.

#### 3.1.2. Follow-Up Data

Follow-up analysis:

- D2 group: Mean follow-up of 3.2 years (range: 0.5–7 years)
- D2plus group: Mean follow-up of 2.1 years (range: 0.2–6 years)

The shorter follow-up duration in the D2plus group represents an important limitation in survival comparisons between groups.

### 3.2. Oncological Outcomes and Survival Rates

#### 3.2.1. Survival Time

**Figure 2:**
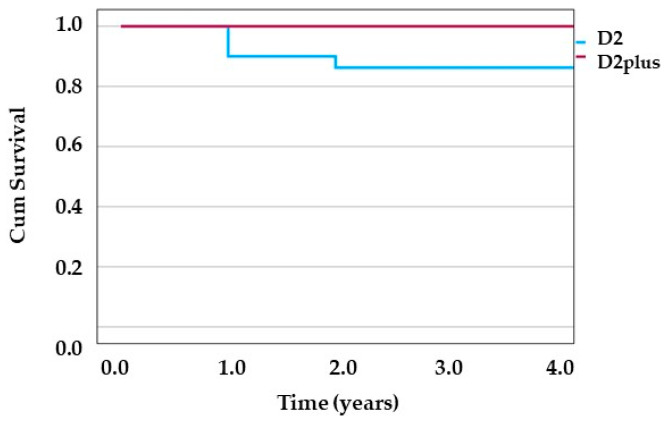
Kaplan-Meier survival curve. Time to event (death) is depicted for D2 group and D2plus group in blue and red line, respectively.

The mean overall survival time in patients undergoing surgery in the D2 group was longer than that of the D2plus group (3.44 ± 2.09 years vs. 2.07 ± 1.65 years, *p* = 0.01). However, this finding must be interpreted with caution due to the significant baseline differences between groups, particularly the higher proportion of SRC in the D2plus group and shorter follow-up duration. The median survival in the D2plus group was one year, while in the D2 group, it was 4 years. Although there were four cancer-related deaths in the D2 group compared to none in the D2plus group during the study period, this difference was not statistically significant (*p* = 0.127, Fisher’s exact test). The Kaplan-Meier survival curve depicting time to death event shows that in the D2 group within the 1st year, three death events occurred, and in the 2nd year, one death event occurred. However, no death events were reported among the D2plus group (Figure 2). The number of deaths was too low for further statistical tests.

#### 3.2.2. Disease Recurrence

A difference in disease recurrence rates was observed between the D2plus group (20.0%) compared to the D2 group (32.4%). However, it was not statistically significant (*p* = 0.29) (Table 1).

#### 3.2.3. The Number of Lymph Nodes Removed During Surgery

In the D2plus group, a mean of 29.4 ± 11.2 lymph nodes was removed, vs. 22.6 ± 8.9 lymph nodes in the D2 group (*p* = 0.013) (Table 1). The number of lymph nodes involved in both groups was low, and no significant difference was found between the groups (*p* = 0.13). Although a greater number of lymph nodes were removed in the D2plus group, no significant increase in survival rates was achieved, raising questions about the clinical utility of extended dissection.

#### 3.2.4. Surgical Complications Rate

The present research findings show that the rate of postoperative complications was significantly higher in the D2plus group (56%, 14/25) compared to the D2 group (20.6%, 7/34, *p* = 0.005) (Table 1). This outcome highlights the surgical challenge involved in extended dissection and the need for an informed selection of patients suitable for the D2plus procedure. Complications were not graded according to the Clavien-Dindo classification due to the retrospective nature of data collection and inconsistent documentation in medical records. Future prospective studies should incorporate standardized complication grading systems. Complications included pancreatic leakage and need for drainage, sepsis, long-term antibiotic treatment, bleeding during surgery, infection of the wound, and one case of mild pancreatitis, which was resolved by conservative treatment. The complications recorded in the D2plus group included mainly inflammations and infections of the pancreas, pancreatic leaks, and massive bleeding during surgery. The risk for complications (gastrointestinal leakage, wound infection, pancreatic leakage, intra-abdominal infection, massive bleeding, pancreatitis) was further assessed through a multivariate analysis (Table 2). The type of dissection, D2plus, increases the risk over five-fold higher than D2 risk for post-surgery complications.

The mean length of hospitalization was similar between the two groups: 18.1 ± 17.3 days in the D2 group compared to 18.6 ± 18.8 days in the D2plus group (*p* = 0.93) (Table 1). This figure indicates that despite the increase in complications in the D2plus group, the length of hospital stay was not significantly different between the groups.

Bivariate analysis showed that preoperative weight loss was found to be significantly associated with post-surgery complications (*p* = 0.043). However, other factors, such as age, gender, smoking, or underlying diseases (diabetes, hypertension, ischemic heart disease), were not found to be significantly related to the rate of surgical complications.

#### 3.2.5. Re-Interventions

The rate of re-interventions (such as drainage, laparotomy, and laparoscopy) 1–14 days after surgery was higher in the D2plus group, with 36% of patients requiring additional procedures compared to 14.7% in the D2 group (*p* = 0.057) (Table 1). Two patients out of three in the D2plus group, who had intra-abdominal infection, required drainage as a reintervention procedure. Although the difference between the groups is not statistically significant, the result indicates an increased need for additional medical care treatment in the D2plus group, which requires clinical caution.

### 3.3. Impact on Quality of Life (QoL)

#### 3.3.1. Weight Loss

The current study data found a higher rate of postoperative weight loss among patients who underwent extended dissection (D2plus) compared to those who underwent standard dissection (D2). Forty percent of the patients in the D2plus group reported weight loss, compared to 17.6% in the D2 group (*p* = 0.056). This may indicate a more significant functional impairment in the D2plus group.

#### 3.3.2. Pain

The percentage of patients who reported pain after surgery was similar in both groups: 70.6% in the D2 group versus 72% in the D2plus group (*p* = 0.91). This figure shows that the post-operative pain does not directly depend on the type of dissection but could be due to general factors related to the nature of the surgery.

### 3.4. Oncological Outcomes

The analysis shows that standard dissection (D2) offers longer overall survival, with a mean survival duration of 3.44 years compared to 2.07 years in the D2plus group (*p* = 0.01). However, this finding must be interpreted with caution due to significant baseline differences, particularly histopathological characteristics. Extended dissection (D2plus) was found to be more effective in reducing disease recurrence rates (20% vs. 32.4% in D2, *p* = 0.29), although the difference was not statistically significant (Table 1). These data indicate the need to customize the surgical approach, depending on the patient’s characteristics and the stage of the disease.

## 4. Discussion

The research findings indicate advantages and disadvantages of extended dissection regarding the D2plus approach in the treatment of stomach cancer patients, yet many questions remain open regarding the effectiveness and safety of this approach.

The higher proportion of signet ring cell carcinoma in the D2plus group (40.0% vs. 20.6%) represents an important confounding factor that may influence survival outcomes. Recent literature by Marano et al. and Roviello et al. has highlighted the significance of SRC percentage as a prognostic factor in gastric cancer, with SRC-predominant tumors often showing more aggressive behavior and poorer prognosis. This histological imbalance between the study groups may partially explain the observed differences in survival outcomes, as SRC histology is associated with worse prognosis regardless of surgical approach [21,22].

The current study finding of shorter mean survival time for D2plus gastric cancer patients is different than that reported by Songun et al. and Dinescu et al., who indicated that the D2 plus approach increases survival rates compared to D2, especially in East Asian countries [3,23]. The possible reasons for this discrepancy could be attributed to more advanced surgical techniques in East Asia. Distinct biology of the disease in different populations, such as West vs. East, which have higher gastric cancer morbidity, could contribute to greater surgical team experience in the latter countries. The study by Basaran et al. showed that there is a clear difference in the benefit of extended dissection between early and advanced stages of gastric cancer [24]. Among patients in early stages (T1), it was found that extensive dissection does not significantly improve survival rates. Of note in the current study, none of the D2plus patients was classified with T1, all patients were M0. These data emphasize the need for a personalized approach when treating gastric cancer patients by the dissection technique, considering the patient’s clinical profile and the stage of the disease [24]. A longer overall survival was observed with the D2plus para-aortic lymph node dissection (PALND) than with D2 for advanced gastric cancer at 5-year survival (49.4% vs. 33% respectively) [8].

The present study data showing low tumor recurrence in patients undergoing D2plus approach is in line with other studies that examined the benefits of extended dissection (D2plus), indicating that this technique can potentially enhance oncological outcomes among gastric cancer patients [3]. Basaran et al. reported that in advanced stages (T2 and above), D2plus contributes to reducing disease recurrence and improving patient survival [24]. These findings support the present study outcomes of the D2plus group, which consisted of patients classified as T2, T3, and T4. Similarly, Liang et al. found a significant decrease in the rate of local–regional disease recurrence in the D2 plus group compared to standard D2 (51.5% vs. 69.5%, *p* < 0.001) [25].

Moreover, the lymph node recurrence rate in the D2 plus group was only 2.3%, compared to 18.7% in the D2 group [25]. These findings support the expanded approach when it comes to advanced cases that require a wider clear margin of the operated tissue area. Therefore, it is important to customize the dissection according to the stage of the disease.

The study of Liang et al. emphasized the benefits of extended dissection among patients with advanced stages of gastric cancer (stages IIIa-b or N1-3a) [25]. The results of the study indicated higher long-term survival rates among those patients who underwent extended dissection (55.3% D2plus versus 43.9% in the D2 group, *p* = 0.042) [25]. As well, Ozmen et al.’s study compared standard D2 dissection and D2 plus para-aortic lymph node (PALN) dissection in patients diagnosed with advanced gastric cancer. The findings support the D2plus PALN approach, which exhibited an increase in survival rates among stage IIIA-IIIB and could be performed without an increase in postoperative morbidity and mortality, and it was safe to be done by experienced surgeons. The findings highlighted the advantage of removing lymph nodes in distant areas in advanced cases, especially in patients with extensive lymphatic involvement.

The main mechanism that explains the benefit of the D2plus approach is the removal of microscopic metastases located in more distant areas, such as the periportal and pancreatic lymph nodes, which are not removed by standard dissection (D2) [26].

The research findings of Wang et al. emphasize the difference in the outcomes of extended dissection (D2 + PAND) compared to standard dissection (D2) depending on the stage of the disease and the characteristics of the patients [27]. The study found that extended dissection does not significantly improve long-term survival rates among all patients but may be beneficial in certain cases of serosa-negative patients [27].

The survival benefits of D2plus are particularly significant in patients with advanced cancer but are sometimes accompanied by an increase in the rate of complications. Therefore, the choice of a personalized approach depends on the clinical characteristics of the patient and the experience of the treating team [26,28].

Increased complication rate with the extended dissection approach was evident in the current study. The multivariate analysis found that the D2plus group had a statistically significantly higher risk than the D2 group (*p* = 0.022) for complications. This outcome is supported by other studies demonstrating that extended dissection (D2plus) involves an increase in surgical complications compared to standard dissection (D2) [9,12,17,25,29]. The serious complications included pancreatic leaks, intraoperative bleeding, and inflammation [30,31]. These findings highlight the surgical challenges involved in extended dissection and the need for careful risk management, especially among patients with risk factors such as underlying diseases or advanced age.

Barone et al. emphasized the importance of considering the balance between standard dissection (D2) and extended dissection (D2plus) in the context of oncological outcomes and surgical complications [29]. The study indicated that standard dissection may be sufficient in some cases, especially in early stages of gastric cancer, where the clinical utility of extended dissection is relatively limited. On the other hand, extended dissection was found to be more effective in advanced cases, but at the cost of a significant increase in the rate of complications and mortality rates [8,32]. Hence, the call for the adoption of an individual approach, based on the evaluation of the stage of the disease and the characteristics of the patients, in order to maximize the oncological benefit while reducing the risk of complications [29,33].

Sasako et al. observed that the complication rates of patients who underwent D2 lymphadenectomy were not statistically different than patients treated by D2 lymphadenectomy plus PAND (20.9% and 28.1% respectively, *p* = 0.07) [34].

One of the decisive factors for the success of an extended dissection and the reduction of the complication rate is the skill level of the surgeons, their training in the complex surgical process, and the experience of the medical center teams, which play a critical role in the surgical outcomes [23,25]. The complication rate of extended dissection is not significantly different from that of standard dissection upon performing the procedure in centers with experienced teams [25,33]. According to Dinescu et al. in East Asia, extended dissection, D2plus, is routinely performed by high-level skilled surgeons, while in the West, the procedure is performed less frequently and in centers with less experience. These data emphasize the need to establish clear protocols and improve surgical training in the West [23].

In the present study, the relatively high gastrointestinal leak rates (8.2% and 20% for D2 group and D2plus, respectively) could possibly reflect technique evolution over a challenging learning curve, where there was a constant shift from open to laparoscopic to robotic techniques. Our current leak rates are consistent with the typical literature benchmarks. Furthermore, the complications recorded in the D2plus group included mainly infections, pancreatic leaks, and massive bleeding during surgery. These findings are consistent with previous studies, which indicated that extended dissection increases the risk of injury to nearby organs and inflammatory processes [35]. However, Ozmen et al. and Yu et al. found that surgical time and complications, such as blood loss, were at a similar rate for D2 and D2plus PALND approaches to gastric cancer surgery [8,36].

Extended dissection (D2 + PAND) requires a longer duration of surgery and was accompanied by more significant blood loss compared to standard dissection [27,34]. Sasako et al. showed that when the D2 + PAND procedure is performed by skilled surgeons, there is no significant increase in post-operative mortality rates [34]. Thus, the recommendation is to prevent complications caused by prolonged surgery time.

The increased reintervention rates observed in the present study among the D2plus approach group reinforce the need to select suitable patients to reduce the risk and to perform the procedure in medical centers with experienced teams.

In the current study, age was not found to be related to surgical complications following extended dissection. The mean age of D2plus group participants was statistically lower than the D2 group (*p* = 0.014). Younger patients, who are relatively healthy, may cope better with the surgical procedure and achieve a significant survival benefit. Hence, they had fewer complications than older patients or those with underlying diseases. The latter group is more likely to develop significant complications after extended dissection [26]. Studies indicate that the patient population in the West, which includes patients with more comorbidities and older age, tends to show worse outcomes following extended dissection compared to outcomes obtained in East Asia. Some argue that these differences may be due to genetic differences, differences in diet, or medical history between the populations [37,38,39,40].

Although a greater number of lymph nodes were removed in the D2plus group of the current study, no significant increase in survival rates was achieved compared to D2 group of patients. These facts raise questions about the clinical utility of extended dissection. Biffi et al.’s study showed that the removal of more than 15 lymph nodes during extended dissection (D2plus) provided a significant survival advantage and a lower rate of distant disease recurrence, even in early cases of the disease (pT1) [33]. These results reiterate the importance of the extended approach even in cases where there is no proven lymphatic involvement because removing more lymph nodes, such as in the periportal and pancreatic regions, improves the staging of the disease and reduces the risk of microscopic metastases located in distant areas [36].

One of the main challenges in the surgical treatment of stomach cancer is the effect of the operation on the patients’ quality of life (QoL), which is affected both by the nature of the disease and the treatment. A multidisciplinary approach based on the patients’ reported outcomes (PROs) allows for a thorough understanding of these effects [28]. PROs provide critical insights into their health and emotional state and could serve as an important tool for planning personalized treatments. In the present study, no significant difference in variables concerning QoL was found in the rate of pain or the duration of hospitalization between the D2 and D2plus groups. However, we acknowledge that the absence of standardized, validated quality-of-life instruments represents a significant limitation of our study. However, in the current study, the quality of life of patients who underwent extended dissection (D2plus) was significantly affected, mainly due to a high rate of weight loss. This outcome is supported by the study of Schütte et al., who reported several symptoms such as weight loss, early satiety, pain, and Post-Gastrectomy Syndrome [28]. These symptoms may last a long time after surgery and affect the ability to lead a normal lifestyle. Despite the negative effects of the surgery, the patients’ quality of life may improve during the 1st year after treatment [28]. It is noted that some symptoms, such as physical fatigue and weight loss, may decrease over time, especially when multidisciplinary support is received [41]. These data underscore the importance of close follow-up and nutritional support after surgery, especially for patients who have undergone extended dissection.

Studies have shown that patients who have undergone extensive surgical operations report a more significant impairment in the quality of life compared to those who have undergone endoscopic treatment in the early stages of the disease [28]. Despite the fact that endoscopic treatment causes fewer physical side effects and enables a faster return to routine, it is sometimes accompanied by increased anxiety about the recurrence of the disease. In contrast, patients who underwent surgical dissection experience greater impairment in physical function.

The scientific literature underlines the need for a balance between the survival benefits of extended dissection and its impact on quality of life [26]. It is noted that extended dissection requires a careful selection of patients to ensure that the oncological benefit justifies the possible damage to daily functioning and general quality of life. The approach of neoadjuvant treatments, using chemotherapy protocols before surgery, brought about a significant change in the way extended dissection is managed. Marano et al. (2023) pointed out that these treatments improve the potential of radical surgery (R0) and reduce microscopic metastases but also cause pathological changes, such as fibrosis, that complicate the dissection process [26]. Combined approaches, such as Neo-D2plus, currently being tested in Italy and Asia, may offer a way to combine the benefits of extended dissection with innovative treatments [42].

Stratifying the different stages makes it possible to avoid unnecessary and complex surgeries in patients who are not expected to benefit significantly while focusing on advanced cases where there is a proven benefit to use the D2plus approach [20,43]. Extended dissection may be warranted in patients with advanced disease. However, physical and mental support should be provided to patients to alleviate adverse effects and improve quality of life after treatment. These findings emphasize the value of extended dissection as part of the oncological treatment in relatively advanced cases but are less relevant in early stages of the disease [25].

A recent study by Kalavacherla et al. compared survival outcomes between minimally invasive gastrectomy to open gastrectomy in treating gastric adenocarcinoma, demonstrating that both gastrectomy types had similar outcomes in overall survival and recurrence-free survival [44].

Study Limitations:

The present study has several important limitations that must be acknowledged:

1. Baseline Imbalances: The significant baseline differences between groups, particularly age (71.6 vs. 63.1 years, *p* = 0.014), gender distribution (70.6% vs. 40% male, *p* = 0.019), and histological characteristics (40% vs. 20.6% SRC), represent important confounding factors that limit the direct comparison of outcomes. Future studies should employ propensity score matching or multivariate adjustment to account for these differences.
2. Sample Size: The relatively small sample size (n = 59) limits statistical power and the ability to detect clinically meaningful differences, particularly in survival outcomes.
3. Retrospective Design: The retrospective nature of data collection introduces potential selection bias and limits the standardization of outcome assessment.
4. Follow-up Duration: The shorter mean follow-up in the D2plus group (2.1 years vs. 3.2 years for the D2 group) may influence survival comparisons and limit long-term outcome assessment.
5. Quality-of-Life Assessment: The absence of standardized, validated quality-of-life instruments represents a significant limitation. Quality-of-life parameters were assessed during routine follow-up visits through clinical evaluation rather than validated questionnaires.
6. Complication Grading: Complications were not graded according to standardized classification systems such as the Clavien-Dindo classification due to inconsistent documentation in retrospective medical records.
7. Single-Center Experience: The study was conducted at a single center, which may limit generalizability to other institutions with different patient populations and surgical expertise. Nevertheless, it reflects valuable data from a retrospective real-world setting.
8. Surgical Experience Variability: During the years 2017–2024, a number of surgeons were performing laparoscopy procedures on gastric cancer patients at Bnai-Zion Medical Center. As this institute is affiliated with the Technion Faculty of Health, training is provided by surgeons, experts in laparoscopy, for medical students, fellows, and interns in the department of surgery. This fact may introduce bias as more than one specialist was operating over the span of 7 years and supervising trainees.

Future research directions could explore the relationship between the number of lymph nodes removed and the effect on oncological outcomes, such as long-term survival rates and disease recurrence rates. These should be further investigated, especially in patients in advanced stages. Further research is needed to improve the understanding of the criteria for choosing the type of dissection and to ensure optimal patient care. In addition, future prospective studies should incorporate standardized complication grading systems.

## 5. Conclusions

The current retrospective study provides preliminary insights into the comparison between D2 and D2plus dissection in a single-center real-world Western cohort. The findings suggest that while D2plus dissection may reduce disease recurrence rates, it is associated with higher complication rates and, in our series, shorter overall survival. The study analysed post-surgery complications, yet the number of deaths (D2 *n* = 4, D2plus *n* = 0) was too low for survival statistical tests. However, the small sample size (*n* = 59), significant baseline differences between groups (particularly age, gender, and histological characteristics), and retrospective design limit our ability to draw definitive conclusions.

The higher proportion of signet ring cell carcinoma in the D2plus group represents a critical confounding factor that may explain survival differences independent of surgical technique. The findings should be interpreted as hypothesis-generating rather than practice-changing.

Future Directions: Larger, prospective, multicenter studies with propensity score matching or randomized design are needed to definitively establish the optimal surgical approach for different patient subgroups. Such studies should incorporate standardized quality-of-life instruments, complication grading systems, and adequate follow-up duration to provide definitive guidance for clinical practice.

It is suggested to adopt a personalized approach to consider the stages of the disease, the characteristics of the patient, and the experience of the surgical team, in addition to focusing on recovery, including nutrition and rehabilitation, to improve the quality of life of patients after gastric cancer surgery. Notwithstanding the oncological advantages of extended dissection, the rate of surgical complications is a major consideration in the decision to perform the procedure. Therefore, performing extended dissections in centers with extensive experience should be prioritized.

## Figures and Tables

**Table 1:** Demographic and clinical characteristics of the study participants.

Characteristic	D2 * N = 34	D2plus * N = 25	Total *N* = 59	*p* Value
Age (years) mean ± SD *	71.6 ± 9.7	63.1 ± 16.2	68.0 ± 16.2	*p* = 0.014
Gender, n (%)				*p* = 0.019
Male	24 (71%)	10 (40%)	34 (58%)	
Female	10 (29%)	15 (60%)	25 (42%)	
Smoking, n (%)	11 (32%)	6 (24%)	17 (28.8%)	*p* = 0.48
Background diseases				
Diabetes, n (%)	7 (20.6%)	8 (32%)	15 (25.4%)	*p* = 0.32
IHD *, n (%)	7 (20.6%)	6 (24)	13 (22%)	*p* = 0.76
HTN *, n (%)	21 (61.8%)	14 (56%)	35 (59.3%)	*p* = 0.66
Hyperlipidemia, n (%)	17 (50%)	10 (40%)	27 (45.8%)	*p* = 0.45
Past abdominal surgery, n (%)	11 (32.4%)	15/24 (62.5%)	26 (44.8%)	*p* = 0.023
Weight loss, n (%)	6 (17.6%)	10 (40%)	16 (27.1%)	*p* = 0.056
Tumor biomarkers				
CA19.9 (units/mL), median [IQR *]	7.2 [3.75–2.05]	12.95 [5.6–20.8]	8 [4.3–20.4]	*p* = 0.38
CA19.9 ^a^, n (%)	27/33 (81.8%)	18/22 (81.8%)	45/55 (81.8%)	*p* = 1.00
CEA * (ng/mL), median [IQR *]	2.5 [1.35–3.35]	2.4 [1.57–3.4]	2.5 [1.5–3.7]	*p* = 0.73
CEA ^b^, n (%)	30 (88.2%)	23 (92%)	53 (89.8%)	*p* = 1.00
Surgical approach				*p* < 0.001
LAP *, n (%)	28 (82.4%)	6 (24%)	34 (57.6%)	
LAP to open, n (%)	1 (2.9%)	3 (12%)	4 (6.8%)	
Open, n (%)	5 (14.7%)	4 (16%)	9 (15.3%)	
Robotic, n (%)	0	12 (48%)	12 (20.3%)	
Surgery type				
Near total gastrectomy, n (%)	4 (11.8%)	6 (24%)	10 (16.9%)	
Subtotal gastrectomy, n (%)	23 (67.6%)	10 (40%)	33 (55.9%)	
Total gastrectomy, n (%)	6 (17%)	9 (36%)	15 (25.4%)	
Wedge, n (%)	1 (2.9%)	0	1 (1.7%)	
Lymphadenectomy				
Dissected lymph nodes, mean ± SD	22.6 ± 8.9	29.4 ± 11.2	25.5 ± 10.4	*p* = 0.013
Invovled lymph nodes, median [IQR *]	0 [0–1.25]	0 [0–0]	0 [0–1]	*p* = 0.13
Invovled lymph nodes, n (%)				*p* = 0.13
N0	20 (58.8%)	20 (80%)	40 (67.8%)	
N1–2	7 (20.6%)	1 (4%)	8 (13.6%)	
N3	7 (20.6%)	4 (16)	11 (18.6%)	
Tumor staging, n (%)				
T1	3 (8.8%)	0	3 (5.1%)	*p* = 0.31
T2	8 (23.5%)	4 (16%)	12 (20.3%)	
T3	22 (64.7%)	19 (76%)	41 (69%)	
T4	1 (2.9%)	2 (8%)	3 (5.1%)	
Neoadjuvant treatment Chemotherapy protocol				
None, n (%)	21 (61.8%)	2 (8%)	23 (39%)	
FLOT, n (%)	12 (35.3%)	18 (72%)	30 (50%)	
FOLFOX, n (%)	1 (2.9%)	5 (20%)	6 (10%)	
Chemotherapy treatment protocol				
None, n (%)	12 (35.3%)	5 (20%)	17 (28.8%)	
FLOT, n (%)	3 (8.8%)	1 (4%)	4 (6.8%)	
FOLFOX, n (%)	9 (26.5%)	4 (16%)	13 (22%)	
Post-surgery				
Pain, n (%)	24 (70.6%)	18 (72%)	42 (71.2%)	*p* = 0.91
Complication, n (%)	7 (20.6%)	14 (56%)	21 (35.6%)	*p = 0.005*
Gastrointestinal leakage	3 (8.2%)	5 (20%)	8 (13.5%)	
Wound infection	2 (5.9%)	4 (16%)	6 (10.2%)	
Pancreatic leakage	1 (2.9%)	1 (4%)	2 (3.4%)	
Intra-abdominal infection	1 (2.9%)	3 (12%)	4 (6.8%)	
Massive bleeding ^c^	0 (0%)	1 (4%)	1 (1.7%)	
Pancreatitis	1 (2.9%)	0	1 (1.7%)	
Reintervention, n (%)	5 (14.7%)	9 (36%)	14 (23.7%)	*p = 0.057*
Gastrointestinal leakage	3 (8.2%)	5 (20%)	8 (13.5%)	
Pancreatic leakage	1 (2.9%)	1 (4%)	2 (3.4%)	
Intra-abdominal infection	1 (2.9%)	3 (12%)	4 (6.8%)	
Hospitalization (days), mean ± SD	18.1 ± 17.3	18.6 ± 18.8	18.3 ± 17.7	*p = 0.93*
EUS *, n (%)	20 (58.8%)	16 (64%)	36 (61%)	*p = 0.68*
Recurrence, n (%)	11 (32.4%)	5 (20%)	16 (27.1%)	*p = 0.29*
Death, n (%)	4 (11.8%)	0	4 (6.8%)	

* Abbreviations: D2 standard gastrectomy dissection; D2plus, extended dissection; SD, standard deviation; IHD, Ischemic Heart Disease; HTN, hypertension; CA19.9, cancer antigen 19.9; CEA, carcinoembryonic antigen; EUS, endoscopic ultrasound; LAP, laparoscopy; IQR, interquartile range; ^a^ CA19.9 values < 30; ^b^ CEA values < 5; ^c^ Massive bleeding > 4 g of hemoglobin loss.

**Table 2:** Multivariate analysis of post-surgery complications.

Variables in the Equation:	B	*p* Value	Odds Ratio	95% CI * Lower	95% CI * Upper
Dissection type ^a^ (1)	1.768	0.022	5.859	1.295	26.511
Gender ^b^ (1)	−0.177	0.791	0.838	0.226	3.106
Age of diagnosis	−0.003	0.910	0.997	0.949	1.048
Weight loss ^c^ (1)	0.811	0.244	2.249	0.575	8.800
Past abdominal surgery ^d^ (1)	−0.432	0.549	0.649	0.158	2.664
Pain ^e^ (1)	1.157	0.142	3.181	0.680	14.875

* Abbreviation: C.I., confidence interval. The categorical variables were coded as follows: ^a^ Dissection type: D2 (0), n = 34; D2plus (1), n = 24. ^b^ Gender: Male (0), n = 34; Female (1), n = 24. ^c^ Weight loss: No (0), n = 32; Yes (1) n = 26. ^d^ Past abdominal surgery: No (0) n = 32; Yes (1) n = 26. ^e^ Pain: No (0) n = 16, Yes (1) n = 42. Of note, one patient had missing data for past surgery. Therefore, n = 58 was the total number of patients included in this statistical multivariate analysis.

## Data Availability

This research data will be made available upon reasonable request from the authors.

## Abbreviations

The following abbreviations are used in this manuscript:

CA	Cancer antigen
CEA	Carcinoembryonic antigen
D2	Standard dissection
D2	Plus extended dissection
EUS	Endoscopic ultrasound
HTN	Hypertension
IHD	Ischemic heart disease
IQR	Interquartile range
LAP	Laparoscopy
PRO	Patient-reported outcome
QoL	Quality of life
SD	Standard deviation
SRC	Signet ring cell carcinoma
